# WNT5A Signaling Contributes to Aβ-Induced Neuroinflammation and Neurotoxicity

**DOI:** 10.1371/journal.pone.0022920

**Published:** 2011-08-17

**Authors:** Bei Li, Ling Zhong, Xiangling Yang, Tommy Andersson, Min Huang, Shao-Jun Tang

**Affiliations:** 1 Department of Neuroscience and Cell Biology, University of Texas Medical Branch, Galveston, Texas, United States of America; 2 School of Pharmaceutical Sciences, Sun Yat-Sen University, Guangzhou, People's Republic of China; 3 Department of Laboratory Medicine, Lund University, Clinical Research Centre, Skåne University Hospital, Malmö, Sweden; University of North Dakota, United States of America

## Abstract

Neurodegenration is a pathological hallmark of Alzheimer's disease (AD), but the underlying molecular mechanism remains elusive. Here, we present evidence that reveals a crucial role of Wnt5a signaling in this process. We showed that Wnt5a and its receptor Frizzled-5 (Fz5) were up-regulated in the AD mouse brain, and that beta-amyloid peptide (Aβ), a major constituent of amyloid plaques, stimulated Wnt5a and Fz5 expression in primary cortical cultures; these observations indicate that Wnt5a signaling could be aberrantly activated during AD pathogenesis. In support of such a possibility, we observed that inhibition of Wnt5a signaling attenuated while activation of Wnt5a signaling enhanced Aβ-evoked neurotoxicity, suggesting a role of Wnt5a signaling in AD-related neurodegeneration. Furthermore, we also demonstrated that Aβ-induced neurotoxicity depends on inflammatory processes, and that activation of Wnt5a signaling elicited the expression of proinflammatory cytokines IL-1β and TNF-α whereas inhibition of Wnt5a signaling attenuated the Aβ-induced expression of the cytokines in cortical cultures. Our findings collectively suggest that aberrantly up-regulated Wnt5a signaling is a crucial pathological step that contributes to AD-related neurodegeneration by regulating neuroinflammation.

## Introduction

Beta-Amyloid (Aβ) peptide is a dominant candidate of the causative agents for Alzheimer's disease (AD) [1], [2]. According to the widely-held amyloid hypothesis of AD, Aβ initiates an array of molecular and cellular cascades that eventually lead to progressive neuronal dysfunction and degeneration [1], [2]. However, mechanistic molecular processes that link Aβ and neurodegeneration remain to be firmly established.

Chronic neuroinflammation associated with persistent glial activation is a major disease process evoked by Aβ and intimately associated with the progress of AD pathologies [3], [4]. Previous studies suggest that neuroinflammation contributes to the development of neurodegenerative hallmarks in AD brains, including Aβ plaques [4] and tau tangles [5], [6], [7]. AD therapeutic approaches that target neuroinflammation are under development [8], [9], [10], [11]. AD neuroinflammation is likely triggered by Aβ-mediated activation of microglia and astrocytes [3], [4], [12],[13],[14]. It was reported that Aβ induces the expression of cytokines (including IL-1β, TNF-α, IL-6, and IL-8) in cultured astrocytes and microglia [15], [16], [17], [18]. Mounting evidence suggests that Aβ may activate glial cells via specific sensor receptors such as toll-like receptors (TLR), receptors for advanced glycoxidation end-products (RAGE) and NOD-like receptors (NLR) [4]. Despite the significant understandings on the induction of AD neuroinflammation, the downstream molecular processes that are elicited by Aβ and regulate the inflammation remain poorly understood.

Wnts are secreted signaling proteins that play important roles in neural development and plasticity [19], [20], [21], [22]. Multiple lines of evidence indicate a critical role of Wnt signaling in AD [22]. β-catenin, a key downstream effector protein in the canonical Wnt signaling pathway, interacts with and is regulated by presenilin [23], [24], [25]. Glycogen synthase kinase (GSK)-3, a central serine/threnine kinase in the canonical Wnt signaling pathway, plays a critical role in the regulation of Aβ production [26] and aggregation [27] and in tau phosphorylation [28]. Genetic studies revealed that LRP6 polymorphisms are causally linked to AD [29]. In AD brains, canonical Wnt signaling is impaired [30], and DKK1, an antagonist of Wnt signaling, is upregulated [31], [32]. Importantly, Aβ was reported to inhibit Wnt signaling by directly binding to the Frizzled receptors [33]. The impairment of canonical Wnt signaling is likely etiologically significant, because forced up-regulation of the canonical Wnt signaling pathway has rescuing effects on the development of AD-related phenotypes in both neuron cultures and animal models [27], [30], [34], [35]. In contrast to the canonical pathway, the involvement of non-canonical Wnt signaling pathways in the regulation of AD pathogenesis is less clear. A recent study indicates that Wnt5a-activated non-canonical Wnt signaling antagonizes Aβ synaptotoxicity [36].

In this paper, we report an important role of Wnt5a signaling in the regulation of Aβ-evoked neurotoxicity and neuroinflammation. We observed that (1) Wnt5a/CaMKII signaling is up-regulated at the early stages of AD development in an APPswe/PSEN1ΔE9 transgenic mouse model, (2) Aβ activates Wnt5a signaling in primary cortical cultures, (3) Aβ-induced Wnt5a up-regulation is a critical molecular step leading to the development of Aβ neurotoxicity in cultures, (4) Wnt5a stimulates inflammatory processes, and (5) Wnt5a is critical for Aβ-induced inflammatory response. Our results suggest that abnormally up-regulated non-canonical Wnt5a signaling may regulate chronic neuroinflammation in AD brains.

## Materials and Methods

### Preparation of Aβ42 peptides

Recombinant human Aβ (1–42) (Chemicon) was used to prepare monomers (Aβ-mon), oligomers (Aβ-olig) and fibrils (Aβ-fib), as described [37]. For monomer preparation, 5 mM Aβ42 in DMSO was diluted directly with cell culture media. For oligomer preparation, 5 mM Aβ42 in DMSO was diluted to 100 µM in ice-cold D-MEM/F-12 (Invitrogen), vortexed for 30″, centrifugated (10,000 g; room temperature) for 1′, and incubated at 4°C for 24 h. For fibril preparation, 10 mM HCl was added to the Aβ42 solution (5 mM in DMSO) to bring Aβ to a final concentration of 100 µM, vortexed for 30″, centrifugated (10,000 g, room temperature) for 1′, and incubated with shaking at 37°C for 24 h. Products from such preparations are mixtures that contain Aβ monomers and the intended aggregates [37]. The quality of the preparations was confirmed by Western blotting analysis with the 6E10 (Covance; 1∶5000), and the molecular mass was estimated by rainbow pre-stained protein markers (GE Healthcare).

### Animals and hippocampus dissection

All procedures were approved by Animal Care and Use Committees of the University of Texas Medical Branch. Male APP (amyloid precursor protein)/presenilin-1 double transgenic (APPswe/PSEN1ΔE9, 2×Tg) mice [38] and wild-type littermates at 3.5 months of age were anesthetized and then decapitated. The hippocampi were rapidly collected on ice for Western blotting, immunohistochemistry, or fluorescent immunostaining..

### Neuron cultures

C57BL E18 cortical cultures were prepared, as described [39]. The cells were plated on poly-D-lysine (30,000–70,000; Sigma) -coated dishes at a density of 1.5×10^5^ cells/cm^2^. Immunostaining with cell-type markers indicate that the cultures are mixtures of neuron (∼60%) and glial cells (∼40%). Cultures at 12–14 DIV were used in this study. All experimental treatments were carried out by adding the administrated agents into the freshly changed culture media. Only morphologically healthy neuronal cultures were used for drug treatments.

### Western blotting and antibodies

Western blotting was performed as described [40]. The intensity of non-saturated bands on Western blots was quantified by densitometry analysis with NIH ImageJ. GAPDH or β-actin were used as loading controls. Rabbit-anti-APP antibody was from Sigma-Aldrich (1∶3000); rabbit-anti-Wnt5a (1∶1000) and rabbit-anti-Fz5 (1∶1000) antibodies were from Abcam; rabbit-anti-p-αCaMKII(Thr286) (1∶1000), rabbit-anti-NIK (1∶1000), and rabbit-anti-IκB-α (1∶1000) antibodies were from Cell Signaling Technology; and mouse-anti-GAPDH (1∶1000) and rabbit-anti-β-actin (1∶1000) antibodies were from Santa Cruz Biotechnology.

### Immunohistochemistry and immunofluorescence

Immunohistochemistry was performed on 5 µm-thick paraffin-embedded sections. The sections were deparaffined, dehydrated and treated with 1% H_2_O_2_ as decribed [41] Antigen retrieval was carried out using citrate buffer (0.01 M; pH 6.0). The sections were then incubated with the primary antibody recognizing Aβ (1–40/42) (Millipore; 1∶100) overnight at 4°C followed by incubation with biotinylated goat anti-rabbit IgG. After treatment with streptavidin-HRP and staining with DAB, the sections were stained with hematoxylin and mounted. Indirect fluorescence immunostaining was performed, as described [42].with rabbit anti-Wnt5a (abcam; 5 µg/ml) and rabbit anti-Fz5 (abcam; 1 µg/ml) primary antibodies and Cy3-conjugated donkey anti-rabbit secondary antibody (Jackson ImmunoResearch; 1∶200).

### Measurement of secreted cytokines

Cortical cultures (12–14 DIV) were treated with Wnt5a (200 ng/ml), and media were collected at 0.5, 1, 2, 6, 12 and 24 h after Wnt5a application. Tumor necrosis factor alpha (TNF-α) and interleukin 1 beta (IL-1β) in collected media were quantified using Mouse TNF-α or IL-1β Quantikine ELISA Kits (R&D Systems), respectively.

### MTT assay and trypan blue staining

The MTT assay and trypan blue staining were performed as described in [43]. Three independent experiments were performed with each experiment including 4–6 replicates for each treatment. Reagents: 3% hydrogen peroxide solution (Sigma-Aldrich); recombinant mouse Wnt-5a (R&D Systems); anti-mouse Wnt-5a antibody (R&D Systems); anti-Fz5 antibody (against the extracellular cysteine-rich domain of human Fz5) (R&D Systems); Foxy5 peptide (Inbiolabs); Box5 peptide (Inbiolabs); recombinant mouse IL-10 (R&D Systems); and activated protein C (APC) (Sigma-Aldrich).

### Statistical analysis

Data were expressed as the means ± SEM. Statistical analysis was performed with the Prism software (GraphPad). We used the Student's two-tailed *t*-test for statistical comparisons between any two groups, and one-way ANOVA analyses with a Bonferroni post-hoc test for comparisons between multiple groups.

## Results

### Increased expression of key proteins in the non-canonical Wnt signaling pathway in the hippocampus of AD mouse models

**Figure 1:**
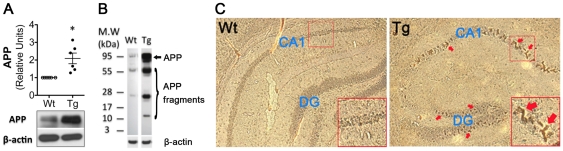
Aβ increases significantly in the hippocampus of the APPswe/PSEN1ΔE9 (2×Tg) mouse at the age of 3.5 months. **A,** APP in the wild-type and 2×Tg hippocampus (3.5-months). B. APP processing in the hippocampus revealed by immunoblotting with the monoclonal antibody 6E10 (recognizing residues 1–16 of Aβ). **C.** Immunohistochemical staining of the hippocampus with 6E10. Note the clustered Aβ signals (arrows) in the cell body layer of the 2×Tg hippocampus. Similar results were obtained from 6 pairs of wild-type and 2×Tg mice.

**Figure 2:**
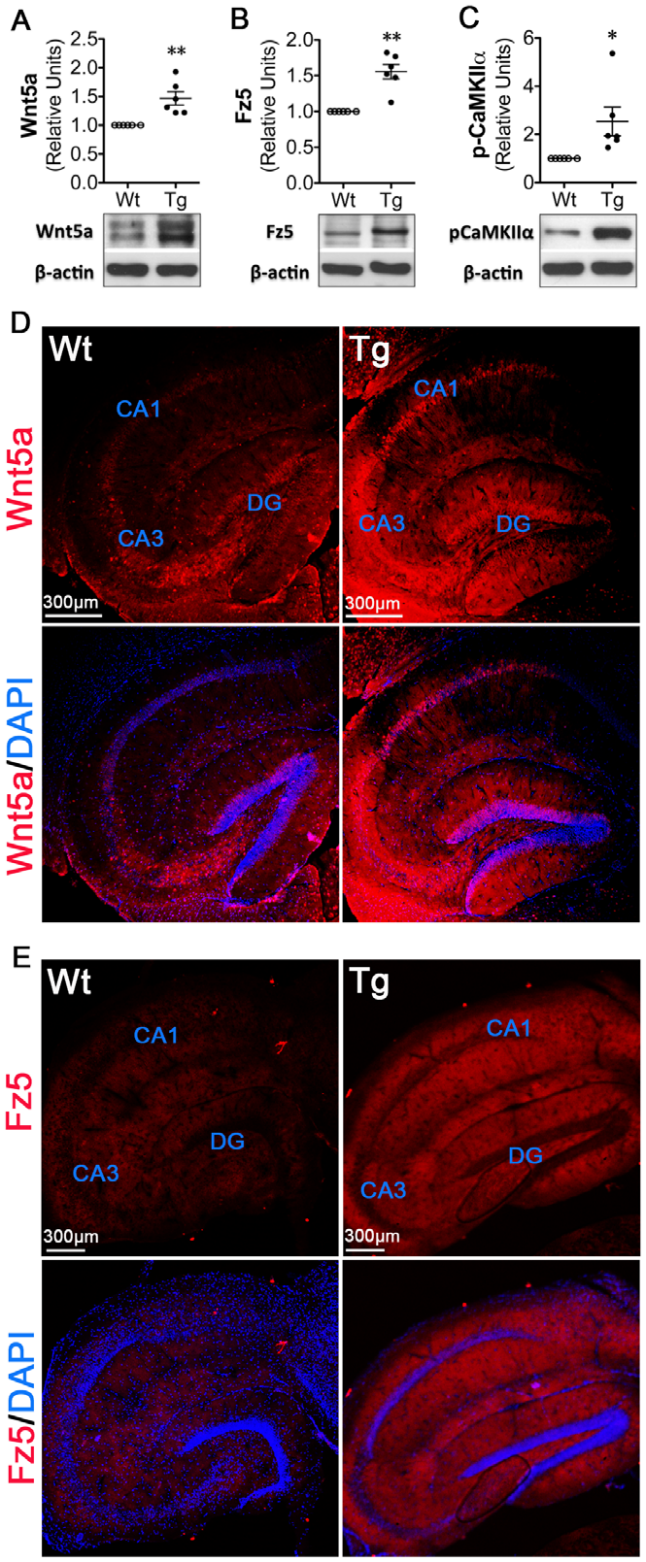
Wnt5a/CaMKII signaling is up-regulated in the hippocampus of the 2×Tg AD mouse model. **A, B,** and **C.** Protein levels of Wnt5a (A), Fz5 (B) and pT286-αCaMKII (C) in the wild-type and 2×Tg hippocampus. In summary graphs of quantitative data, the levels of target proteins were normalized with the β-actin loading control and expressed as relative units to the wild-type controls. Data presented in graphs are means ± SEM from six pairs of mice. * *p*<0.05. **D** and **E.** Distribution of Wnt5a (D) and Fz5 (E) protein in the wild-type and 2×Tg hippocampus. DAPI staining was performed to visualize the cell bodies.

Previous studies revealed down-regulation of canonical Wnt signaling in the brain of APPswe/PSEN1ΔE9 AD mouse models [27]. Consistent with these findings, we observed decreased expression of proteins in the canonical pathway, including Wnt3a, Fz1 and β-catenin (data not shown). In contrast, in preliminary studies, we also observed up-regulated expression of several proteins implicated in the non-canonical Wnt signaling pathway, including Wnt5a and its receptor Fz5, at various postnatal stage (3.5–9.5 months). At 3.5 months of age, APP transgene was clearly expressed (Fig. 1A). APP processing expected to generate Aβ was evident in the transgenic hippocampi (Fig. 1B). In addition, immunohistochemistry staining with an antibody that recognized Aβ revealed clustered signals in the cell body layer (Fig. 1C), which may include Aβ that starts accumulating. Because memory deficits have not manifested at this stage [44], we reasoned that the up-regulation of non-canonical Wnt signaling is potentially an early molecular abnormality during AD pathogenesis. Therefore, we performed more detailed analysis on the non-canonical Wnt signaling in mice at 3.5 months of age. Western blotting analysis showed that, compared with wild-type controls, Wnt5a protein increased 1.5 fold in the hippocampus of the 2×Tg mouse (*p*<0.01; Fig. 2A). Similarly, Fz5 protein was also significantly up-regulated in the 2×Tg hippocampus (1.6 fold; *p*<0.01; Fig. 2B). Because activation of non-canonical Wnt signaling can cause αCaMKII phosphorylation [45], we next determined the protein level of phosphorylated αCaMKII (pT286-αCaMKII). pT286-αCaMKII was increased 2.6 fold (*p*<0.05; Fig. 2C). Fluorescent immunostaining showed that, in comparison with control, the increase of Wnt5a (Fig. 2D) and Fz5 (Fig. 2E) occurred in all hippocampal fields, including CA1, CA2/CA3 and dentate gyrus (DG), of the 2×Tg mouse, which resembled the spatial pattern of Aβ plaques (Fig. 1C). These data together suggest that non-canonical Wnt signaling is up-regulated in the 2×Tg AD hippocampus.

### Rapid up-regulation of non-canonical Wnt signaling by Aβ oligomers in primary neuron culture

**Figure 3:**
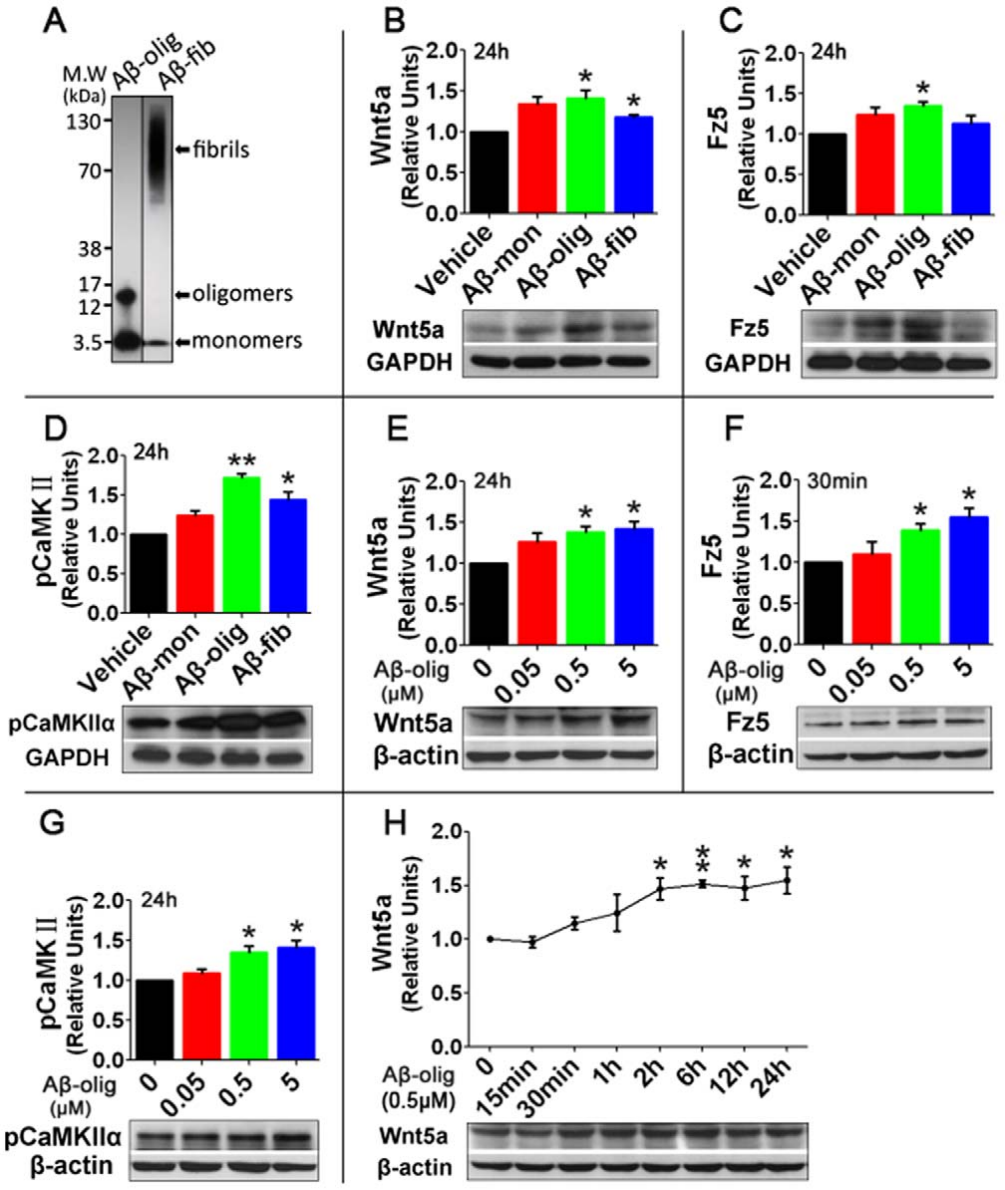
Up-regulation of proteins in the non-canonical Wnt signaling pathway by Aβ peptide in cortical neuron cultures. **A.** Western blot analysis of the preparations of Aβ (1–42) oligomers and fibrils. Aβ preparations were, separated on SDS–PAGE (4–12% NuPAGE bis-Tris gel), and probed with the monoclonal antibody 6E10.. **B, C,** and **D.** Protein levels of Wnt5a (B), Fz5 (C) and pT286-αCaMKII (D) in cortical cultures treated with 500 nM of monomeric, oligomeric, and fibrillar Aβ peptide for 24 hrs. **E, F,** and **G.** Concentration-dependent effects of Aβ oligomer on the protein levels of Wnt5a (E), Fz5 (F) and pT286-αCaMKII (G). Although the dose effect of Aβ on Wnt5a was most clearly observed at 24 hours post Aβ administration, the effect on Fz5 could be observed at 30 min. **H.** Time course of the Aβ oligomer effects (500 nM) on Wnt5a. In summary graphs of quantitative data, the levels of target proteins were normalized against GAPDH or β-actin and expressed as relative units to the vehicle-treated controls. Data in summary graphs are means ± SEM from three independent experiments. * *p*<0.05; ** *p*<0.01.

We next sought to understand the mechanism by which non-canonical Wnt signaling is activated in the AD mouse brain. Because Aβ is a key pathological agent for AD, we hypothesized that it causes the up-regulated expression of Wnt5a and other relevant proteins measured in 2×Tg mice. To test this hypothesis, we determined the effect of Aβ on the expression of these proteins in mouse cortical cultures (12–14 DIV). A wide range of Aβ concentrations (100 nM - 15 µM) have been used in previous *in vitro* studies. We chose to use a relatively low Aβ concentration (500 nM) to better reflect the fact that the proteins are up-regulated at early AD stages when Aβ begins to accumulate (Fig. 1C). Prior works have shown that 500 nM Aβ is efficient to initiate some of the earliest and reversible AD-relevant pathologies [46], [47]. More recent studies have indicated that different forms of Aβ (monomer, oligomer and fibril) vary in their AD-related toxicity, with the oligomer being considered the most toxic form [37], [48]. We prepared the oligomers and fibrils of Aβ (1–42) (Fig. 3A) and compared the effect of Aβ monomers, oligomers and fibrils on Wnt5a, Fz5 and pT286-αCaMKII protein expression in cortical cultures. As shown in Fig. 3B–D, Aβ oligomer treatment for 24 h caused significant increases in Wnt5a (1.4 fold; *p*<0.05), Fz5 (1.4 fold; *p*<0.01) and pT286-αCaMKII (1.7 fold; *p*<0.01). Under the same experimental conditions, preparations of monomers and fibrils caused changes with lower magnitudes (Fig. 3B–D). Next, we investigated the dose effects of the oligomer. As shown in Fig. 3E–G, the oligomer increased expression of Wnt5a, Fz5, and pT286-αCaMKII proteins in a concentration-dependent manner (50 nM–5 µM). Although Wnt5a and p-CaMKII displayed a clear dose-effect at 24 hours after treatment, Fz5 showed this effect at 30 min. In addition, 500 nM of Aβ oligomer caused a time-dependent increase in Wnt5a (Fig. 3H). These results demonstrated that the non-canonical Wnt5a signaling is up-regulated by Aβ peptide, especially the oligomers.

### Suppression of Wnt5a signaling alleviates Aβ neurotoxicity

**Figure 4:**
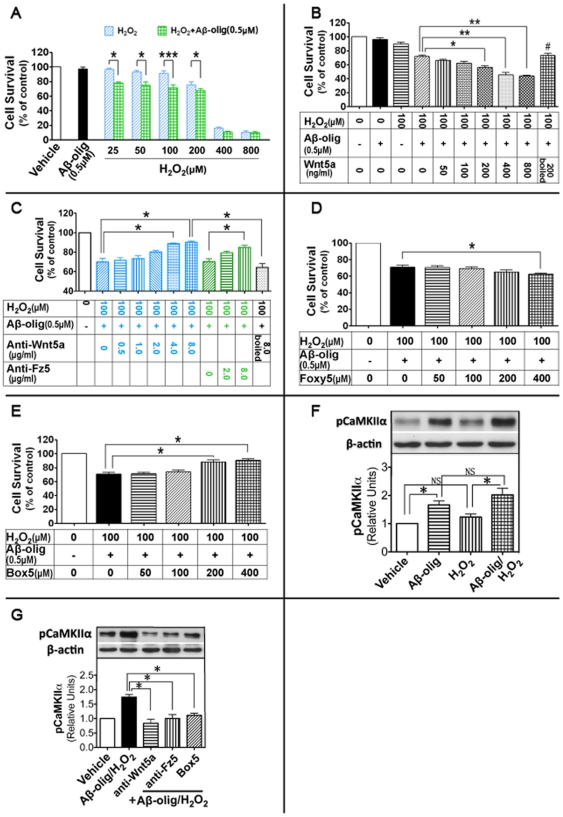
Wnt5a signaling regulates Aβ neurotoxicity. The MTT assay was used to measure cell viability in cortical cultures at 24 hrs after indicated treatments. **A.** Cell survival rates in cultures challenged with Aβ oligomers, H_2_O_2_, or a combination of Aβ oligomers and H_2_O_2_. **B.** Cultures challenged with Aβ (500 nM) and H_2_O_2_ (100 µM) in the presence of Wnt5a at different concentrations. **C.** Cultures challenged with Aβ and H_2_O_2_ in the presence of anti-Wnt5a or ant-Fz5 antibodies. **D.** Effects of Foxy5 on the neurotoxicity of Aβ/H_2_O_2_. **E.** Effects of Box5 on the neurotoxicity of Aβ/H_2_O_2_. Data are expressed as mean ± SEM (n≥12 from three independent experiments). * *p*<0.05; ** *p*<0.01; *** *p*<0.001. # *p*<0.05 (vs. the group of Wnt5a (200 ng/ml)+Aβ/H_2_O_2_). **F.** Protein levels of pT286-αCaMKII in cortical cultures treated with Aβ (500 nM), H_2_O_2_ (100 µM), or the combination of Aβ and H_2_O_2_. **G.** Protein levels of pT286-αCaMKII in cortical cultures treated with anti-Wnt5a (4 µg/ml), anti-Fz5 (4 µg/ml) antibodies or Box5 (200 µM), in the presence of Aβ (500 nM) and H_2_O_2_ (100 µM). * *p*<0.05; NS, not significant.

The observations of the upregulation of the Wnt5a signaling in early AD mouse brains and by Aβ in cortical cultures suggest a potential role of Wnt5a signaling in AD pathogenesis. We next sought to directly test this idea by determining the significance of Aβ-mediated Wnt5a up-regulation on Aβ cytotoxicity in cortical cultures. Previous work revealed concentration-dependent neurotoxicity of Aβ peptides [37], [49], [50], with high concentrations of Aβ (>5.0 µM) causing neuron death in cultures and low concentrations of Aβ (0.1∼1.0 µM) sensitizing neurons to become vulnerable to further stresses [50]. To mimic the early AD brain condition when the non-canonical Wnt signaling proteins are aberrantly upregulated, in these experiments we used a relatively low concentration (500 nM) of Aβ that can upregulate Wnt5a and Fz5 (Fig. 3). Aβ oligomer at this concentration did not cause cell death, as measured by MTT assays (Fig. 4A). We also used trypan blue staining to confirm this observation (Fig. S1). This finding is consistent with the idea that in the early stages of AD pathogenesis Aβ may not cause neuron death. Next, we wanted to investigate if Aβ at this concentration causes the cells to become more vulnerable to detrimental stresses that are relevant to AD pathogenesis. To this end, we tested the effect of Aβ on the sensitivity of the cultures to hydrogen peroxide (H_2_O_2_) stresses. Hydrogen peroxide was chosen because it is generated during the very early stages of aggregation of the amyloid peptides [51] and is critically involved in AD pathogenesis [49], [52]. At low concentrations (25–100 µM), hydrogen peroxide did not cause cell death by itself; however, when co-administrated with Aβ oligomers (500 nM) it caused marked cell death (Fig. 4A, Fig. S1). These results indicate that neurons exposed to 500 nM Aβ are more vulnerable to other detrimental factors. We also showed that H_2_O_2_ neither stimulated αCaMKII phosphorylation by itself nor significantly enhanced the Aβ-induced phsphorylation (Fig. 4F), indicating that H_2_O_2_ did not activate the non-canonical pathway.

To determine the potential role of Wnt5a up-regulation in Aβ-induced sensitization to H_2_O_2_ stress, we neutralized Wnt5a in media with specific anti-Wnt5a antibody. As shown in Fig. 4C, the antibody (0.5–8.0 µg/ml) displayed a rescue effect on cell death in a concentration-dependent manner, which reached statistical significance after 4.0 µg/ml. Recent studies identified a modified Wnt5a-derived hexapeptide (Box5) that can specifically antagonize Wnt5a in malignant melanoma cells [53]. Similar to the anti-Wnt5a antibody, Box5 also had a rescue effect on Aβ/H_2_O_2_-induced cell death in a concentration-dependent manner (Fig. 4E). Because Fz-5 is a Wnt5a receptor [54], we further tested the effect of anti-Fz5 antibody (against the extracellular cystein-rich domain). Consistent with the findings with anti-Wnt5a antibody and Box5, the anti-Fz5 antibody also had a rescue effect (Fig. 4C). We further tested the effect of, anti-Wnt5a, anti-Fz5 antibodies and Box5 on the phosphorylation of αCaMKII, a molecular marker of the activation of the non-canonical Wnt/Ca^2+^ pathway. The results showed that all these reagents significantly blocked Aβ/H_2_O_2_-induced up-regulation of p-αCaMKII (Fig. 4G), and thus provide an independent molecular confirmation of the activity of anti-Wnt5a, anti-Fz5 antibodies and Box5. These results together strongly suggest that Aβ-induced Wnt5a up-regulation at least partially mediates Aβ neurotoxicity.

### Exogenous Wnt5a potentiates Aβ neurotoxicity

Because Aβ-induced Wnt5a up-regulation is critical for Aβ neurotoxicity (Fig. 4C and E), we next investigated if exogenous Wnt5a can potentiate Aβ/H_2_O_2_-induced cell injury. To this end, we tested the effect of recombinant Wnt5a on Aβ/H_2_O_2_ neurotoxicity. As shown in Fig. 4B, compared with Aβ/H_2_O_2_-treated cultures, addition of purified recombinant Wnt5a protein (50–800 ng/ml) increased cell death in a concentration-dependent manner. This potentiation activity of Wnt5a was completely abolished when the protein was heat-inactivated (Fig. 4B). This observation indicates that the biochemical activity of Wnt5a rather than the mere presence of Wnt5a peptide is essential for the potentiation. In addition, Foxy5, a Wnt5a-derived hexapeptide (the same peptide as Box5 but differently modified) that can mimic Wnt5a-induced activities in malignant melanoma and in breast cancer cells [55], was also able to potentiate the neurotoxicity of Aβ/H_2_O_2_ (Fig. 4D). These results indicate that Wnt5a is sufficient to elicit neurotoxicity.

### Anti-inflammation factors attenuate Aβ neurotoxicity

**Figure 5:**
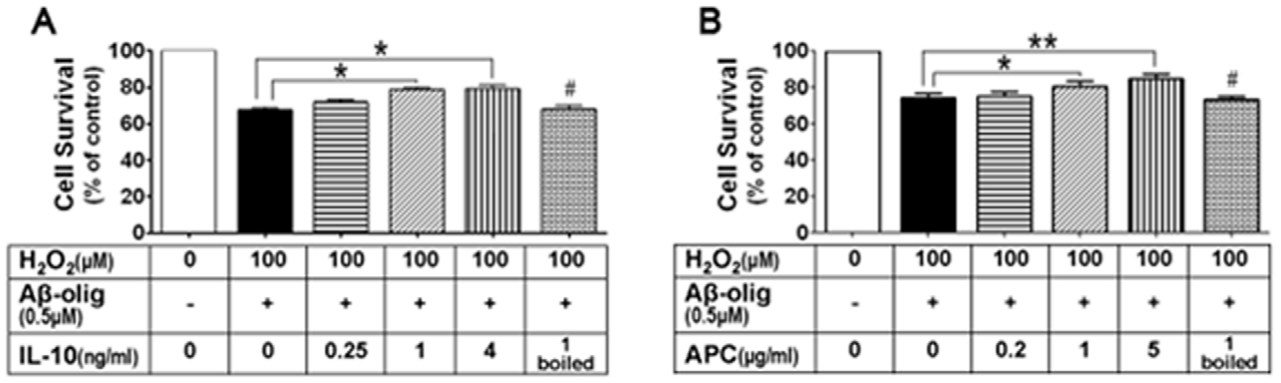
Anti-inflammatory factors alleviate Aβ neurotoxicity. **A.** Effects of IL-10 on cell viability of cortical cultures challenged with Aβ/H_2_O_2_ for 24 hrs. **B.** Effects of APC. Data (means ± SEM) are from three independent experiments. * *p*<0.05; ** *p*<0.01; # *p*<0.05 (vs. the group treated with active IL-10 (1 ng/ml) or APC (1 µg/ml) in the presence of Aβ/H_2_O_2_).

The results described above indicate that up-regulated Wnt5a may mediate Aβ neurotoxicity. We then sought to understand the mechanism by which Wnt5a contributes to Aβ neurotoxicity. Because recent studies revealed a critical role of Wnt5a in the regulation of inflammatory responses in peripheral systems, we were interested in determining if Wnt5a contributes to Aβ neurotoxicity by regulating inflammation. To this end, we first investigated the contribution of neuroinflammation to the Aβ-induced sensitization of cortical cultures to H_2_O_2_ stress. We found that IL-10, a prototypic anti-inflammatory cytokine, caused concentration-dependent rescue effects on Aβ toxicity (Fig. 5). IL-10 started to rescue Aβ/H_2_O_2_-induced cell death at 0.25 ng/ml and reached a plateau effect at 1 ng/ml (Fig. 5A). Heat-inactivated IL-10 did not have any rescuing effect (Fig. 5A). To further confirm the involvement of inflammation in the Aβ toxicity, we determined the effect of activated protein C (APC), another well established anti-inflammatory protein. The results showed that APC displayed a similar rescue effect as IL-10 (Fig. 5B). These results indicate that neuroinflammatory response likely contributes to Aβ neurotoxicity.

### Wnt5a regulates proteins that involve in inflammatory response in cortical cultures

**Figure 6:**
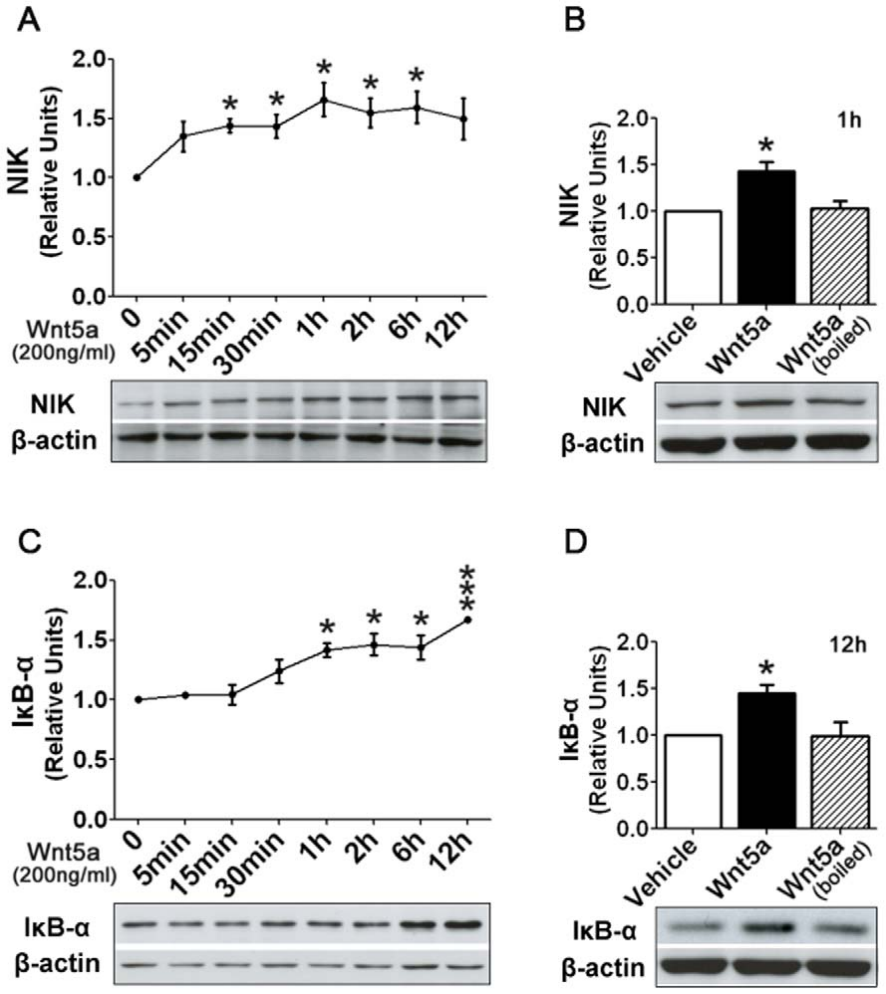
Wnt5a activates molecular pathways critical for inflammatory response. **A.** The time course of NIK protein dynamics after Wnt5a (200 ng/ml) treatment. **B.** Heat-inactivated Wnt5a cannot induce NIK. **C.** The time course of IκB-α protein dynamics after Wnt5a (200 ng/ml) treatment. **D.** Heat-inactivated Wnt5a cannot induce of IκB-α protein. * *p*<0.05; *** *p*<0.001 (vs. 0 h in **A** and **C**, or vs. vehicle in **B** and **D**).

Recent studies reveal an important role of Wnt5a in control of inflammatory response in non-neuronal peripheral tissues [56], [57], [58], but its involvement in regulation of inflammatory response in nervous systems has not been reported. To investigate the potential contribution Wnt5a signaling to neuroinflammation, we examined the effect of Wnt5a on the protein expression of NF-κB-inducing kinase (NIK), a key positive regulator of inflammation that controls NF-κB activity by promoting the processing of p100, the inactive NF-κB2 precursor, to produce the functional p52 subunit [59]. We observed that NIK protein began to increase 5 min after Wnt5a administration (200 ng/ml), and this increase continued gradually, peaked at 60 min. and stayed at a higher level afterward (Fig. 6A). Heat-inactivated Wnt5a did not induce a NIK increase (Fig. 6B), indicating that Wnt5a protein activates inflammatory pathways in cortical cultures. We also investigated the effect of Wnt5a on the expression of IκB-α protein, an inhibitory regulator of inflammation response by sequestering NF-κB in the cytosol [60]. Interestingly, Wnt5a also induced a gradual increase of IκB-α (Fig. 6C). In contrast to the NIK increase, the IκB-α up-regulation did not begin until 30–60 min after Wnt5a treatment but continued for the rest of the time that measurements were recorded (Fig. 6C). It is possible that the lag in up-regulation of IκB-α functions to resolve the Wnt5a-initiated inflammatory response.

**Figure 7:**
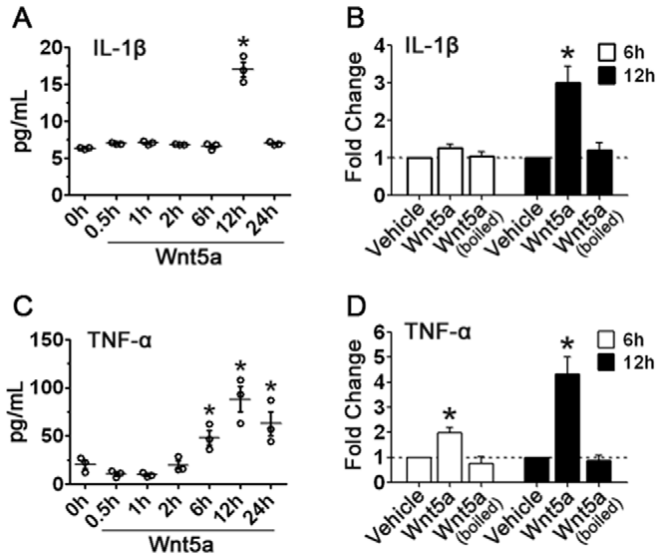
Wnt5a evokes cytokine release. **A.** The temporal profiles of IL-1β in media of Wnt5a-treated cortical cultures measured by ELISA. **B.** Heat-inactivation abolished the activity of Wnt5a in eliciting IL-1β release. **C.** The temporal profiles of TNF-α in media after Wnt5a treatment. **D.** Effects of heat-inactivation on the Wnt5a activity in eliciting TNF-α release. * *p*<0.05 (vs. 0 h in **A** and **C**, or vs. vehicle in **B** and **D**).

Inflammatory response culminates with the secretion of an array of inflammatory cytokines. If Wnt5a indeed induces the inflammatory response, specific inflammatory cytokines must be secreted after Wnt5a administration. Thus, we used ELISA to measure IL-1β and TNF-α in culture media after Wnt5a treatment for 0.5, 1, 2, 6, 12, or 24 hrs. We observed that Wnt5a stimulation caused a 2.7-fold increase in IL-1β at 12 h, while no change was detected at other time points (Fig. 7A). On the other hand, TNF-α increased significantly at 6 h (2.3 fold), 12 h (4.3 fold) and 24 h (3.1 fold) (Fig. 7C). However, this effect of Wnt5a was completely abolished when the protein was heat-inactivated (Fig. 7B and D). These results together strongly suggest that Wnt5a can elicit the inflammatory response in neuron cultures.

### Blockage of Wnt5a signaling impairs Aβ-induced up-regulation of proinflammatory cytokines

**Figure 8:**
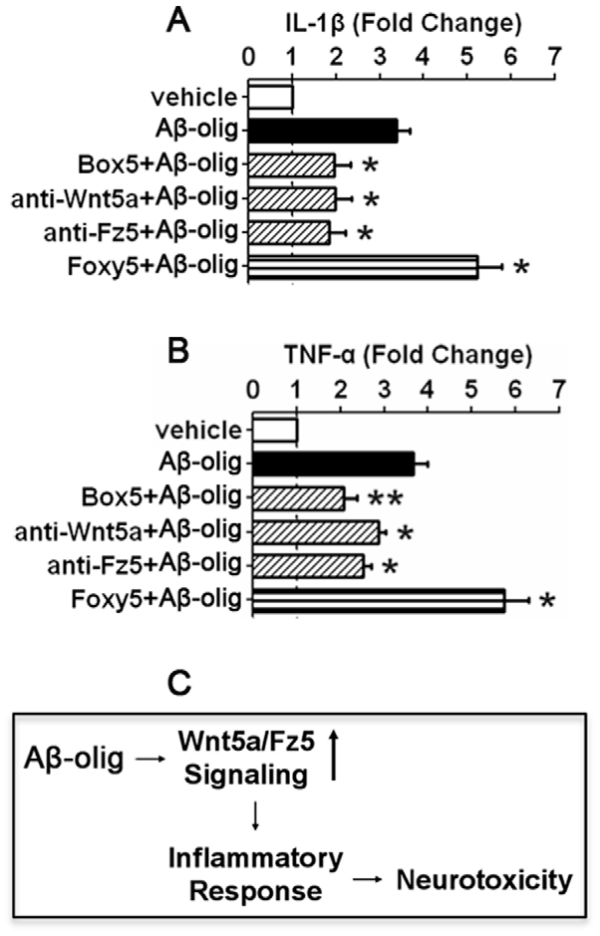
Wnt5a is critical for Aβ-elicited cytokine release. **A.** Inhibition of Wnt5a/Fz5 signaling impaired Aβ-evoked IL-1β increase in media. **B.** Inhibition of Wnt5a/Fz5 signaling impaired Aβ-evoked TNF-α increase in media. ELISA assay of IL-1β (**A**) and TNF-α (**B**) secreted in cortical cultures treated for 12 hrs with Aβ (500 nM) or Aβ and Box5 (200 µM), anti-Wnt5a (2 µg/ml) or anti-Fz5 (2 µg/ml) antibody. Foxy5, a Wnt5a agonist, potentiates the cytokine release. * *p*<0.05; ** *p*<0.01 (vs. vehicle). **C.** A working model for the role of Wnt5a signaling in AD pathogenesis.

Because Aβ evoked inflammation-mediated neurotoxicity, we next investigated the effect of Wnt5a signaling inhibition on the Aβ-elicited inflammatory response. As shown in Fig. 8A, Aβ oligomers (500 nM) significantly increased IL-1β release (3.4 fold; p<0.05). Box5 peptide (200 µM), anti-Wnt5a (2 µg/ml) or anti-Fz5 (2 ug/ml) antibody suppressed the increase of IL-1β induced by Aβ, by 58%, 59% and 55%, respectively (all 3 with p<0.05). In contrast, Foxy5 (200 µM), an agonist of Wnt5a, increased IL-1β release (1.5 fold, compared with Aβ alone; p<0.05; Fig. 8A). Box5, anti-Wnt5a and anti-Fz5 antibodies caused similar effects on Aβ-induced TNF-α release (Fig. 8B). These results together demonstrate that the suppression of Wnt5a signaling inhibits Aβ-evoked inflammatory responses in cortical neuron cultures.

## Discussion

In this study, we found that non-canonical Wnt5a signaling is up-regulated in mouse brains prior to AD phenotypes and by Aβ peptide in cortical neuron cultures. The up-regulated Wnt5a signaling contributes to the inflammation-dependent Aβ neurotoxicity in cultures. We also found that Wnt5a up-regulates inflammation regulatory proteins and proinflammatory cytokines and that Wnt5a is required for the Aβ-induced proinflammatory cytokines. These observations collectively suggest the following working model (Fig. 8C): accumulation of Aβ in the brain aberrantly up-regulates Wnt5a signaling, which in turn evokes an inflammatory response that causes neurodegeneration or cell death in AD brains.

The observed up-regulation of Wnt5a signaling is probably an early etiologically relevant event during AD development. Both Wnt5a and Fz5 proteins significantly increase in the APPswe/PSEN1ΔE9 hippocampus at the age of 3.5 months (Fig. 2). Previous studies showed that this AD mouse model started to accumulate Aβ plaques after 4 months of age [61] and did not develop cognitive impairments until 5–7 months of age [44]. Thus, the observed Wnt5a and Fz5 up-regulation at 3.5 months of age is likely prior to the development of major AD phenotypes. This notion is consistent with the finding that a relatively low concentration of Aβ is able to up-regulate Wnt5a and Fz5 (Fig. 3), suggesting that Wnt5a signaling is a potential target for slowing or blocking early AD pathogenesis.

Converging lines of evidence support a critical role of the down-regulation of the canonical Wnt/β-catenin pathway in AD pathogenesis. In contrast, the involvement of non-canonical Wnt signaling is less clear. Our findings reveal an early up-regulation of Wnt5a signaling in the hippocampus of 2×Tg AD mice. Etiological significance of this dysregulation is suggested by the observation that Wnt5a signaling is necessary for Aβ to fully induce neurotoxicity in cortical cultures. Previous studies demonstrated that down-regulation of canonical signaling contributed to Aβ neurotoxicity [27], [30], [34], [62]. It is possible that Aβ causes parallel up-regulation of the non-canonical Wnt signaling and down-regulation of the canonical signaling to initiate neurotoxicity cascades. The Wnt canonical and non-canonical pathways often antagonize one another [63]. Thus, another possible scenario is that Aβ may directly down-regulate the canonical pathway, as suggested by a recent study [33], which consequently causes the up-regulation of the non-canonical pathway.

Our results reveal a neurotoxic activity of Wnt5a signaling, and this Wnt5a activity contributes to Aβ toxicity in neuron cultures. Cerpa et al. recently reported that acute administration of exogenous Wnt5a (500 nM) prevented Aβ-induced synaptotoxicity within 40 min after Aβ oligomer application [36]. Their results indicate a synapto-protective activity of Wnt5a signaling soon after Aβ exposure. Because Aβ-up-regulated Wnt5a does not occur by 1 hour after Aβ treatment in cultures (Fig. 3H) and 500 nM Aβ itself does not induce obvious cell death in this period (Fig. 4A and S1), we reason that basal Wnt5a has a synapto-protective activity. On the other hand, sustained up-regulation of Wnt5a, which occurs at 2 hours after Aβ treatment (Fig. 3H), probably potentiates neurotoxicity.

We further found that activation of Wnt5a signaling stimulates the expression of proinflmmatory cytokine in cortical cultures (Figs. 6 and 7). This finding indicates that up-regulation of Wnt5a may mediate Aβ-induced neuroinflammation in AD brains (Fig. 8C). Because the Aβ-elicited inflammatory response (Fig. 8A, 8B) and alleviated Aβ-induced neurotoxicity (Fig. 4) was impaired by the anti-Wnt5a antibody and Box5, Aβ likely induces Wnt5a secretion, although the kinetics of the secretion is currently unknown. In peripheral non-neuronal systems, Wnt5a is implicated in inflammation of multiple chronic disorders, including rheumatoid arthritis [64], sepsis [56], atherosclerosis [65], melanoma [66], and psoriasis [67]. Our results provide the initial evidence for a critical role of Wnt5a signaling in the regulation of inflammatory responses in CNS disorders. Because the primary cortical cultures used in this study contain neurons and glia (including microglia and astrocytes), we currently do not know the specific type of glial cells through which Wnt5a evokes the observed inflammatory responses. In a recent study, Halleskog et al. reported that Wnt3a stimulated the expression of proinflammatory cytokines in microglia [68]. It would be interesting to know if Wnt5a regulates neuroinflammation by stimulating the same or different types of glia. Nonetheless, the findings on Wnt5a and Wnt3a indicate that proteins in the Wnt family may orchestrate neuroinflammatory response during AD pathogenesis.

## Supporting Information

Figure S1 **Cell survival rates revealed by trypan blue staining.** Cortical cultures at 24 hrs after indicated treatments were used. Dying cells were stained due to the increase of membrane permeability to the dye.
(TIF)
