# Supplementary figures and images for: WNT5A Signaling Contributes to Aβ-Induced Neuroinflammation and Neurotoxicity
Source: PLoS One. 2011 Aug 17;6(8):e22920. doi: 10.1371/journal.pone.0022920 (PMC3157339; doi:10.1371/journal.pone.0022920)

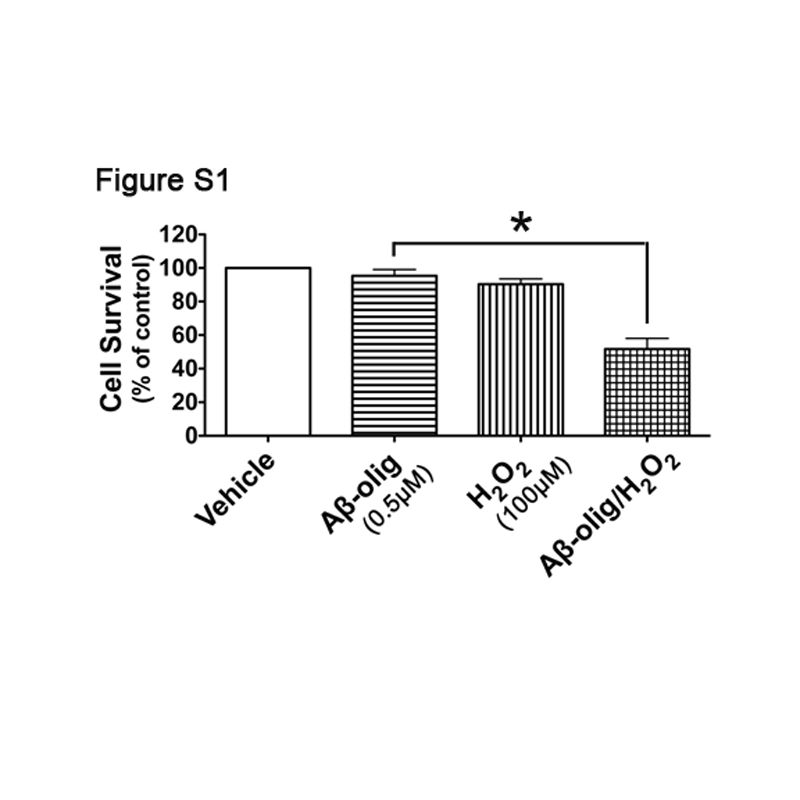

Supplement: Figure S1 — Cell survival rates revealed by trypan blue staining. Cortical cultures at 24 hrs after indicated treatments were used. Dying cells were stained due to the increase of membrane permeability to the dye. (TIF) [file pone.0022920.s001.tif]
